# hsa_circ_0001955 Enhances *In Vitro* Proliferation, Migration, and Invasion of HCC Cells through miR-145-5p/NRAS Axis

**DOI:** 10.1016/j.omtn.2020.09.007

**Published:** 2020-09-16

**Authors:** Bisha Ding, Weimin Fan, Weiyang Lou

**Affiliations:** 1 Department of Breast Surgery, The First Affiliated Hospital, College of Medicine, Zhejiang University, 79 Qingchun Road, Hangzhou, Zhejiang 310003, China; 2 Program of Innovative Cancer Therapeutics, Division of Hepatobiliary and Pancreatic Surgery, Department of Surgery, First Affiliated Hospital, College of Medicine, Key Laboratory of Combined Multi-Organ Transplantation, Ministry of Public Health, Key Laboratory of Organ Transplantation, Zhejiang University, Hangzhou 310003, Zhejiang, China

**Keywords:** circular RNA, circRNA, hsa_circ_0001955, hepatocellular carcinoma, HCC, microRNA, miRNA, miR-145-5p, NRAS

## Abstract

Increasing circular RNAs (circRNAs) have been reported to act as key players in human malignancies. However, the expression, role, and mechanism of circRNAs in HCC are not well elucidated. In this study, some differentially expressed circRNAs (DECs) between hepatocellular carcinoma (HCC) and normal tissues were identified using three circRNA microarrays (Gene Expression Omnibus [GEO]: GSE78520, GSE94508, and GSE97332). Twenty-one DECs were found to be commonly upregulated in all the three datasets. Among the 21 DECs, hsa_circ_0001955 ranked as the top three most upregulated DECs in GEO: GSE78520, GSE94508, and GSE97332. Moreover, hsa_circ_0001955 expression in HCC cells and tissues was significantly higher than that in corresponding normal controls. Functional experiments revealed that knockdown of hsa_circ_0001955 markedly inhibited proliferation, migration, and invasion of HCC, and its overexpression led to the opposite effects. hsa_circ_0001955 was mainly located in the cytoplasm, in which hsa_circ_0001955 could directly bind to miR-145-5p. miR-145-5p was downregulated in HCC, and its expression was negatively linked to hsa_circ_0001955 expression. Furthermore, we identified that NRAS was a downstream direct target of the hsa_circ_0001955/miR-145-5p axis in HCC. Collectively, our findings demonstrate the oncogenic roles of the hsa_circ_0001955/miR-145-5p/NRAS axis in HCC, which may represent a potential therapeutic target for HCC.

## Graphical Abstract

**Figure:**
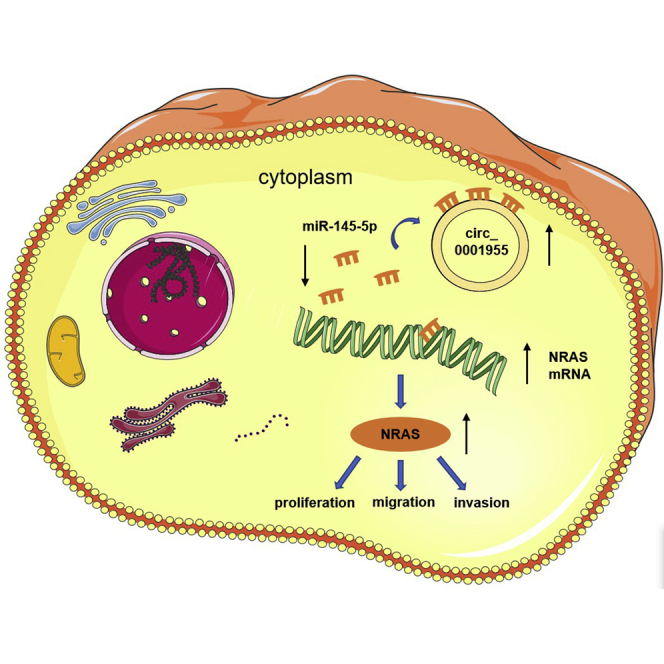

## Teaser

The findings demonstrate the oncogenic roles of the hsa_circ_0001955/miR-145-5p/NRAS axis in HCC, which may represent a potential therapeutic target for HCC.

## Introduction

Liver cancer is one of the leading causes of cancer-associated mortality all over the world. Hepatocellular carcinoma (HCC) is the most common type of primary liver cancer, accounting for approximately 80% of all cases.^1^ To date, multiple risk factors have been found to link to HCC occurrence and development, such as virus infection,^2^ alcohol abuse,^3^ non-alcoholic fatty liver disease,^4^ and cirrhosis.^5^ Additionally, several lines of evidence have indicated that accumulation of genetic and epigenetic changes leads to HCC.^6^

Circular RNAs (circRNAs) are a class of endogenous non-coding RNAs, with covalently closed continuous loop structures.^7^ circRNAs are usually generated from exons of back-splicing protein-coding genes, thus making them more stable than their linear transcripts.^8^ circRNAs are abundantly expressed in various organisms and play crucial roles in a variety of biological processes through acting as competitive endogenous RNAs (ceRNAs) of miRNAs to regulate target gene expression.^9^ Growing studies have well documented that circRNAs are frequently dysregulated in many human disorders, including cancer.^10^, ^11^, ^12^ Recently, some circRNAs have been reported to participate in HCC onset and progression. For example, Liu et al.^13^ suggested that circRNA-5692 inhibited HCC progression by sponging miR-328-5p to promote DAB2IP expression, Wang et al.^14^ reported that SOX9-induced circ-FOXP1 enhanced HCC progression via sponging miR-875-3p and miR-421, and Su et al.^15^ found that circRNA Cdr1as functioned as a ceRNA to promote HCC progression. However, the knowledge of circRNA’s function and mechanism in HCC remains insufficient and needs to be further investigated.

To further explore the expression, function, and mechanism of circRNAs in HCC, we obtained differentially expressed circRNAs (DECs) between HCC and normal tissues by analyzing circRNA microarray data from three Gene Expression Omnibus [GEO] datasets, including GEO: GSE78520, GSE94508, and GSE97332. Among all DECs, we noticed that hsa_circ_0001955 ranked in the top three most upregulated DEC sets of all three datasets. We also confirmed that hsa_circ_0001955 expression was dramatically upregulated in HCC cell lines and tissues compared with corresponding normal controls, and found that its high expression was significantly positively correlated with large tumor size and advanced TNM stage. Moreover, functional experiments demonstrated that hsa_circ_0001955 facilitated proliferation, migration, and invasion of HCC cells. Mechanistically, our results showed that hsa_circ_0001955 increased NRAS expression by sponging miR-145-5p, thereby leading to growth and metastasis of HCC. Taken together, we illustrated that hsa_circ_0001955 functioned as an oncogenic circRNA by affecting the miR-145-5p/NRAS axis in HCC, which may provide a promising target for HCC therapy in the future.

## Results

### hsa_circ_0001955 Was Identified as the Potential circRNA in HCC

**Figure 1:**
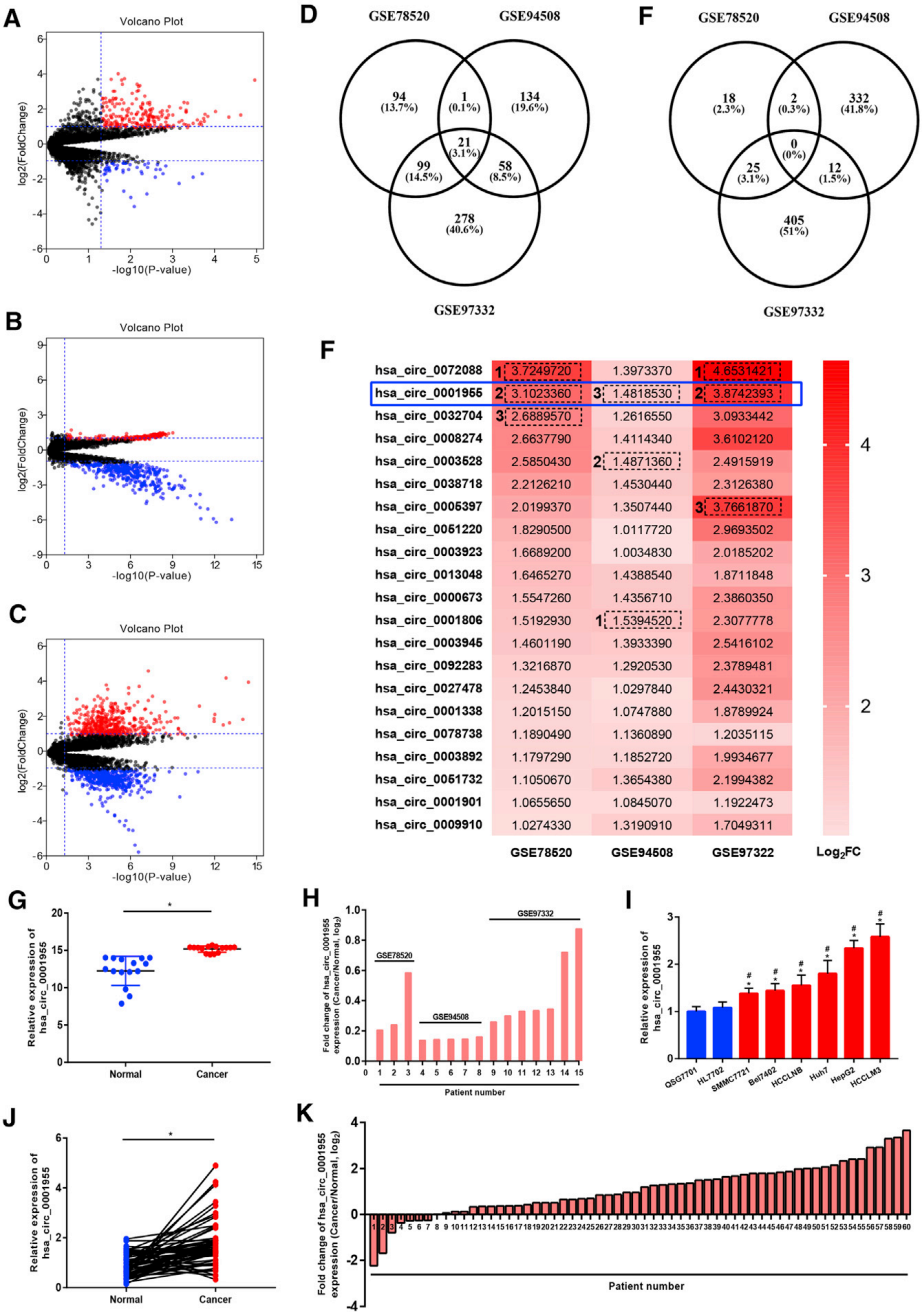
Identification of hsa_circ_0001955 as the Most Potential circRNA in HCC (A) The volcano plot of DECs in GSE78520. (B) The volcano plot of DECs in GSE94508. (C) The volcano plot of DECs in GSE97332. The red and blue dots represented upregulated DECs and downregulated DECs with statistical significance (|FC| > 2 and p < 0.05), respectively. (D) The DECs were commonly upregulated in GEO: GSE78520, GSE94508, and GSE97332. (E) The DECs were commonly downregulated in GEO: GSE78520, GSE94508, and GSE97332. (F) The heatmap of 21 DECs that were commonly upregulated in GEO: GSE78520, GSE94508, and GSE97332. (G) hsa_circ_0001955 expression was significantly increased in HCC tissues compared with normal tissues from GEO: GSE78520, GSE94508, and GSE97332. ∗p < 0.05. (H) The expression change (cancer/normal) of hsa_circ_0001955 in 15 HCC patients from GEO: GSE78520, GSE94508, and GSE97332. (I) hsa_circ_0001955 expression in HCC cell lines was higher than that in normal liver cell lines. ∗p < 0.05 (compared with QSG7701); ^#^p < 0.05 (compared with HL7702). (J) hsa_circ_0001955 was markedly upregulated in HCC tissues compared with normal tissues. ∗p < 0.05. (K) The expression change (cancer/normal) of hsa_circ_0001955 in 60 HCC patients.

To find the potential circRNAs in HCC, we used the GEO database in this work. By a series of selection procedures as we mentioned below, three circRNA microarray datasets, including GSE78520, GSE94508, and GSE97332, were finally included. GEO2R analysis for the three datasets demonstrated that a number of circRNAs were significantly aberrantly expressed in HCC (filtered by |fold change [FC]| ≥ 2 and p < 0.05) (Figures 1A–1C), and these circRNAs were renamed as significant DECs. All significant DECs from GEO: GSE78520, GSE94508, and GSE97332 were listed in Tables S1, S2, and S3, respectively. Next, we intersected the significant upregulated and downregulated DECs from GEO: GSE78520, GSE94508, and GSE97332 to further obtain some DECs with more interest. As shown in Figure 1D, 21 significant DECs were commonly upregulated in GEO: GSE78520, GSE94508, and GSE97332. However, none of these significant DECs were commonly downregulated in all three datasets (Figure 1E). For better visualization, the FC heatmap of 21 commonly upregulated DECs was presented in Figure 1F. Based on FC, hsa_circ_0072088, hsa_circ_0001955, and hsa_circ0032704 were the top 3 most upregulated in GSE78520; hsa_circ_0001806, hsa_circ_0003528, and hsa_circ0001955 were the top 3 most upregulated in GSE94508; and hsa_circ_0072088, hsa_circ_0001955, and hsa_circ0005397 were the top 3 most upregulated in GSE97332. We found that, among these circRNAs, only hsa_circ_0001955 was commonly presented in the top 3 most upregulated DEC sets in GEO: GSE78520, GSE94508, and GSE97332. Thus, in the following studies, we focused on the circRNA has_circ_0001955. As depicted in Figures 1G and 1H, hsa_circ_0001955 expression in the 15 HCC samples from GEO: GSE78520, GSE94508, and GSE97332 was higher than that in 15 normal samples. Moreover, we experimentally confirmed that hsa_circ_0001955 expression was markedly increased in HCC cell lines and clinical samples compared with normal liver cell lines and matched adjacent normal tissues (Figures 1I–1K). Additionally, we also analyzed the correlation of hsa_circ_0001955 expression with various clinicopathological characteristics of HCC patients (Table 1). The result revealed that the expression level of hsa_circ_0001955 was significantly associated with large tumor size (p = 0.0027) and advanced TNM stage (p = 0.0063). Taken together, hsa_circ_0001955 was identified as a potential circRNA in HCC.

**Table 1:** The Correlation between hsa_circ_0001955 Expression and Clinicopathological Features in 60 HCC Patients

	All Cases	hsa_circ_0001955 Expression		
Features	Total	Low	High	p Value
Total number	60	26	34	
Age at surgery (years)				0.8752
>55	27	12	15	
≤55	33	14	19	
Gender				0.8315
Male	36	16	20	
Female	24	10	14	
HBV infection				0.0859
Absent	26	8	18	
Present	34	18	16	
Liver cirrhosis				0.1344
With	32	11	21	
Without	28	15	13	
Tumor size (cm)				0.0027
>5	28	6	22	
≤5	32	20	12	
TNM stage				0.0063
I + II	35	10	25	
III + IV	25	16	9	

### hsa_circ_0001955 Facilitated Proliferation, Migration, and Invasion of HCC

**Figure 2:**
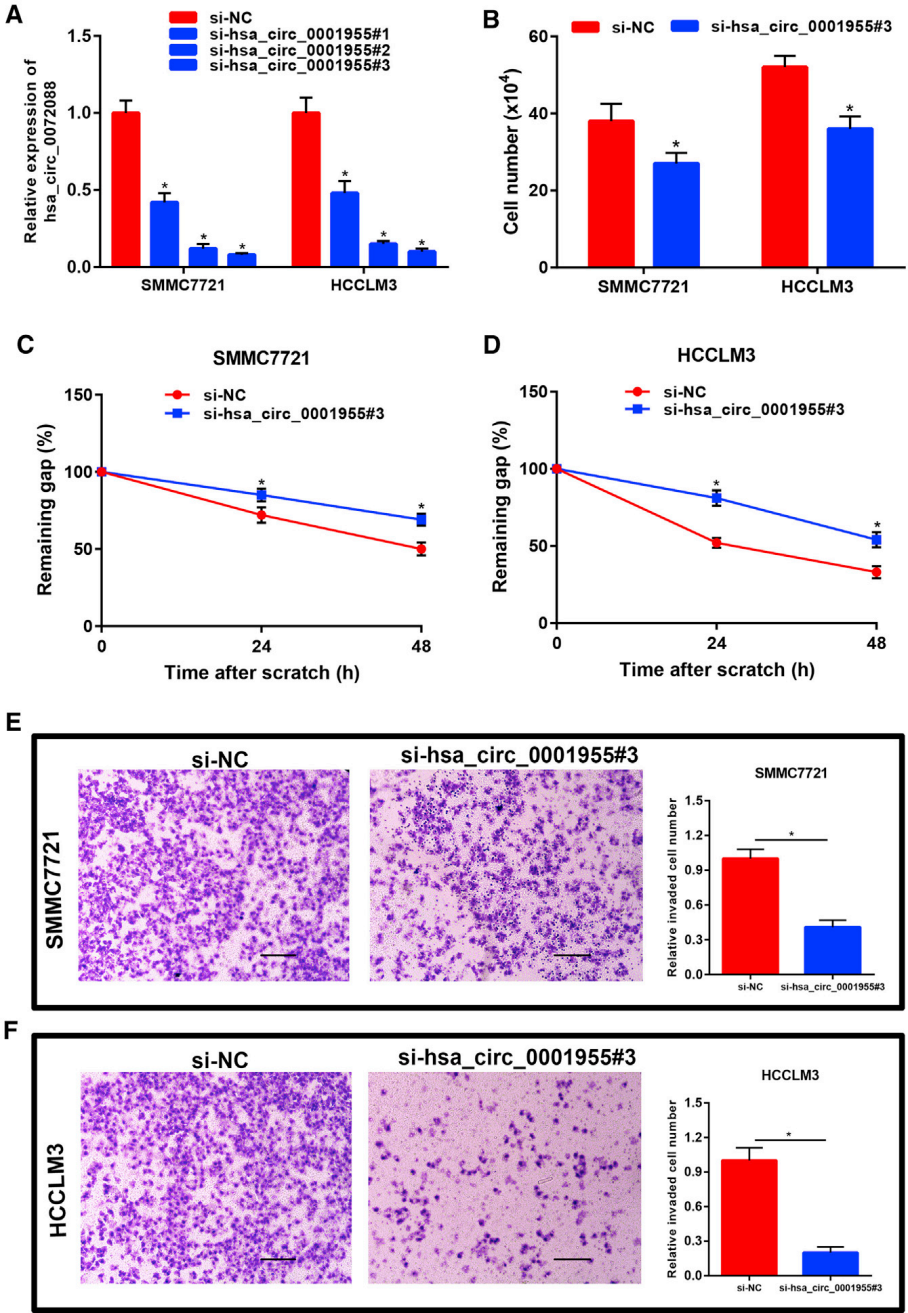
Knockdown of hsa_circ_0001955 Suppressed Proliferation, Migration, and Invasion of HCC (A) The knockdown effect of siRNAs targeting hsa_circ_0001955 in SMMC7721 and HCCLM3. (B) Silencing of hsa_circ_0001955 inhibited cell growth of HCC. (C) Suppression of hsa_circ_0001955 expression weakened SMMC7721 migration. (D) Suppression of hsa_circ_0001955 expression weakened HCCLM3 migration. (E) Knockdown of hsa_circ_0001955 impeded SMMC7721 invasion. (F) Knockdown of hsa_circ_0001955 impeded HCCLM3 invasion. Scare bars: 100 μm. ∗p < 0.05.

**Figure 3:**
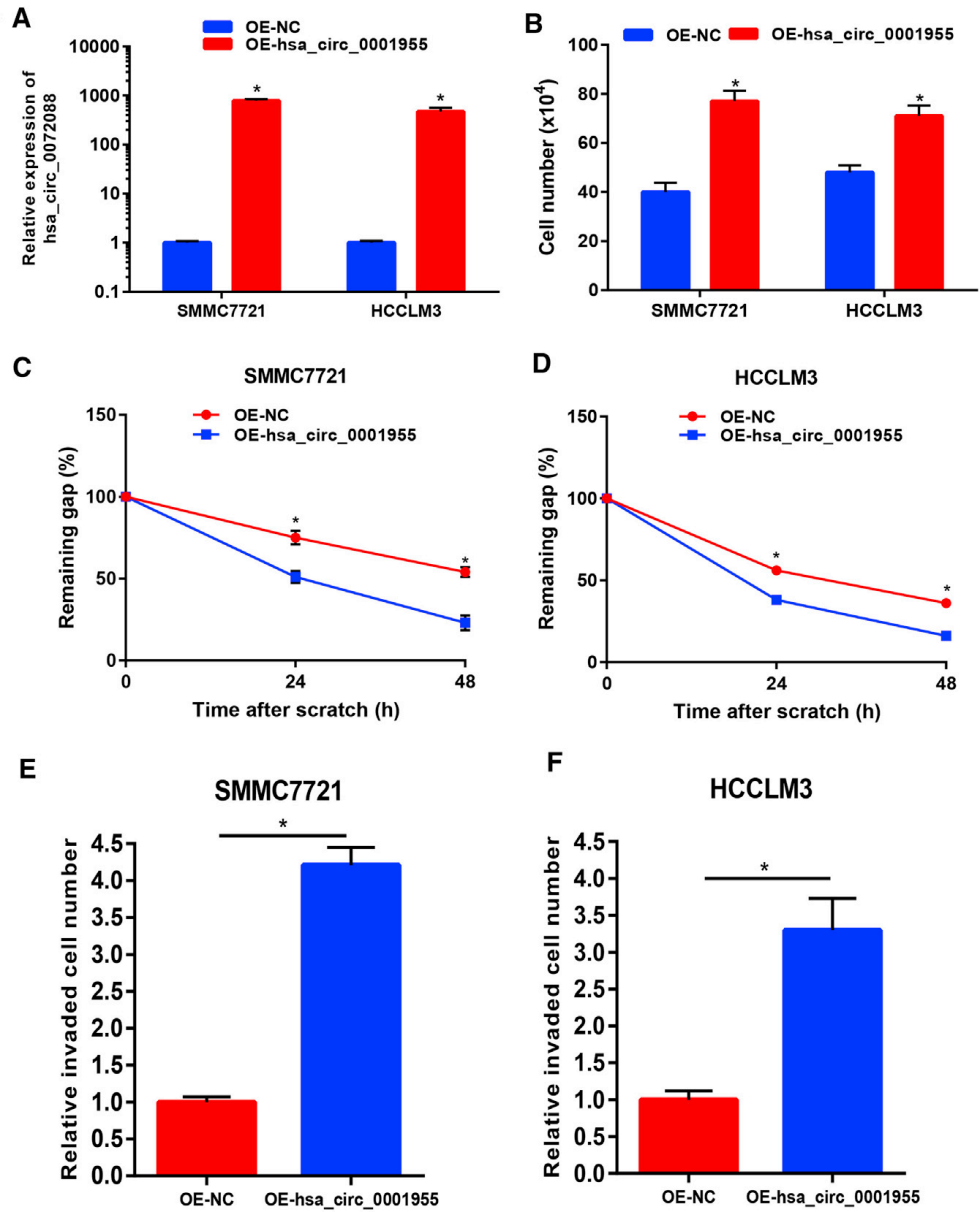
Overexpression of hsa_circ_0001955 Promoted Proliferation, Migration, and Invasion of HCC (A) The overexpressed effect of plasmid carrying hsa_circ_0001955 in SMMC7721 and HCCLM3. (B) Upregulation of hsa_circ_0001955 enhanced cell growth of HCC. (C) Upregulation of hsa_circ_0001955 expression enhanced SMMC7721 migration. (D) Upregulation of hsa_circ_0001955 expression enhanced HCCLM3 migration. (E) Increased expression of hsa_circ_0001955 facilitated SMMC7721 invasion. (F) Increased expression of hsa_circ_0001955 facilitated HCCLM3 invasion. ∗p < 0.05.

The above results together demonstrated that hsa_circ_0001955 might act as an oncogenic circRNA in HCC. Thus, we intended to explore its functions in HCC. Considering the high expression level of hsa_circ_0001955 in HCC, we first used the small interfering RNA (siRNA) knockdown method. Three siRNAs were designed to target the unique back-splicing junction of hsa_circ_0001955. SMMC7721 with the lowest expression of hsa_circ_0001955 and HCCLM3 with the highest expression of hsa_circ_0001955 were employed as two represented cell lines in this study. As shown in Figure 2A, the three siRNAs significantly decreased hsa_circ_0001955 expression in both SMMC7721 and HCCLM3 cells. Among the three siRNAs, the third siRNA, hsa_circ_0001955#3, possessed the best knockdown effect, and it was used to perform the following functional experiments. Downregulation of hsa_circ_0001955 markedly inhibited SMMC7721 and HCCLM3 cell growth (Figure 2B). Wound healing assay and transwell invasion assay also suggested that silencing of hsa_circ_0001955 significantly suppressed cell migration (Figures 2C and 2D) and invasion (Figures 2E and 2F). Next, hsa_circ_0001955-overexpressing plasmid was constructed and employed to increase hsa_circ_0001955 expression (Figure 3A). Figure 3B showed that cell growth capability was obviously enhanced after upregulation of hsa_circ_0001955. Overexpression of hsa_circ_0001955 also promoted SMMC7721 and HCCLM3 migration and invasion as shown in Figures 3C–3F. Collectively, these findings indicated that hsa_circ_0001955 functioned as an oncogenic circRNA in controlling proliferation, migration, and invasion of HCC cells.

### Identification of miR-145-5p as the Direct Binding miRNA of hsa_circ_0001955 in HCC

**Figure 4:**
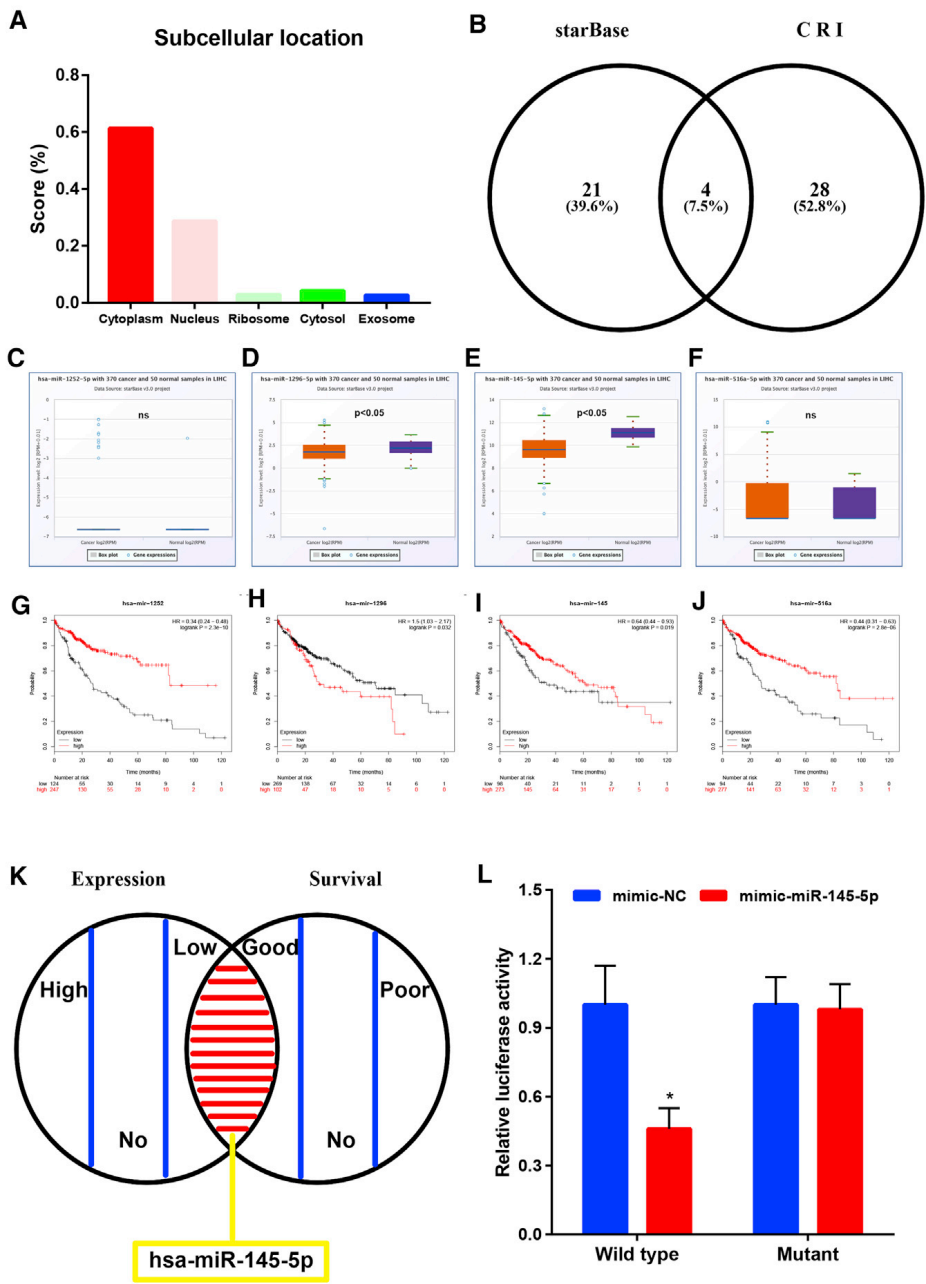
miR-145-5p Was a Binding miRNA of hsa_circ_0001955 in HCC (A) hsa_circ_0001955, with the most possibility, located in cytoplasm predicted by lncLocator. (B) Four common miRNAs (miR-1252-5p, miR-1296-5p, miR-145-5p, and miR-516a-5p) of hsa_circ_0001955 were predicted by starBase and CRI (Circular RNA Interactome). (C) The expression of miR-1252-5p in HCC. (D) The expression of miR-1296-5p in HCC. (E) The expression of miR-145-5p in HCC. (F) The expression of miR-516a-5p in HCC. (G) The prognostic value of miR-1252-5p in HCC. (H) The prognostic value of miR-1296-5p in HCC. (I) The prognostic value of miR-145-5p in HCC. (J) The prognostic value of miR-516a-5p in HCC. (K) miR-145-5p was identified as the most potential miRNA of hsa_circ_0001955 by combination of expression and survival analyses. (L) The direct relationship between miR-145-5p and hsa_circ_0001955 was determined by dual-luciferase reporter assay. ∗p < 0.05. ns, represented no significant difference.

As reported, cytoplasmic circRNAs may act as miRNA sponges to exert their roles. To explore the possibility and ability of hsa_circ_0001955 to bind to miRNAs in HCC, the potential subcellular location of hsa_circ_0001955 was first predicted by an online tool named lncLocator (http://www.csbio.sjtu.edu.cn/cgi-bin/lncLocator.py). As presented in Figure 4A, hsa_circ_0001955 was forecasted to mainly locate in the cytoplasm with the highest predicted score (0.62). Next, two online bioinformatics databases (starBase and Circular RNA Interactome [CRI]) were introduced to find potential miRNAs binding to hsa_circ_0001955. Eventually, only four miRNAs, including miR-1252-5p, miR-1296-5p, miR-145-5p, and miR-516a-5p, were commonly predicted by both starBase and CRI databases (Figure 4B). Subsequently, we assessed their expression levels (Figures 4C–4F) and prognostic values (Figures 4G–4J) in HCC using TCGA data. Based on the miRNA sponge mechanism of circRNA and the oncogenic role of hsa_circ_0001955 in HCC, the potential binding miRNAs of hsa_circ_0001955 should be tumor-suppressive miRNAs in HCC. Among the four miRNAs, only miR-1296-5p and miR-145-5p were significantly downregulated in HCC tissues when compared with normal tissues. Survival analysis demonstrated that high expression of miR-1252-5p, miR-145-5p, and miR-516a-5p had favorable prognosis in HCC. By combination of expression analysis and survival analysis, we supposed that miR-145-5p was the most potential miRNA that may be sponged by hsa_circ_0001955 (Figure 4K), which was later validated by a dual-luciferase reporter assay (Figure 4L). Collectively, these results revealed that miR-145-5p could directly bind to hsa_circ_0001955 in HCC.

### miR-145-5p Played Tumor-Suppressive Roles and Rescued hsa_circ_0001955’s Effects on HCC

**Figure 5:**
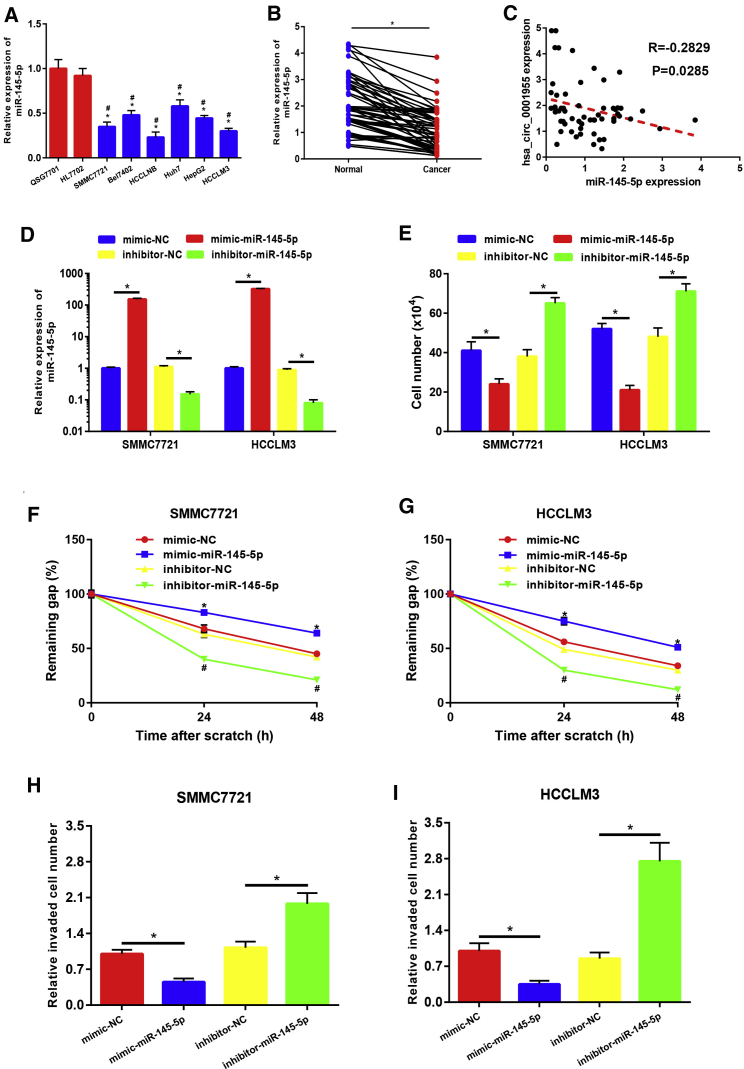
miR-145-5p Played Tumor-Suppressive Roles in HCC (A) miR-145-5p was significantly downregulated in HCC cell lines compared with normal liver cell lines. (B) miR-145-5p expression in HCC tissues was lower than that in normal tissues. (C) miR-145-5p expression was negatively correlated with hsa_circ_0001955 expression in 60 HCC tissues. (D) Transfection effect of miR-145-5p mimic and inhibitor in SMMC7721 and HCCLM3. (E) Overexpression of miR-145-5p suppressed cell growth, whereas knockdown of miR-145-5p promoted cell growth in HCC. (F) Overexpression of miR-145-5p suppressed SMMC7721 migration, whereas knockdown of miR-145-5p promoted migration. (G) Overexpression of miR-145-5p suppressed HCCLM3 migration, whereas knockdown of miR-145-5p promoted migration. (H) Overexpression of miR-145-5p suppressed SMMC7721 invasion, whereas knockdown of miR-145-5p promoted invasion. (I) Overexpression of miR-145-5p suppressed HCCLM3 invasion, whereas knockdown of miR-145-5p promoted invasion. ∗p < 0.05.

**Figure 6:**
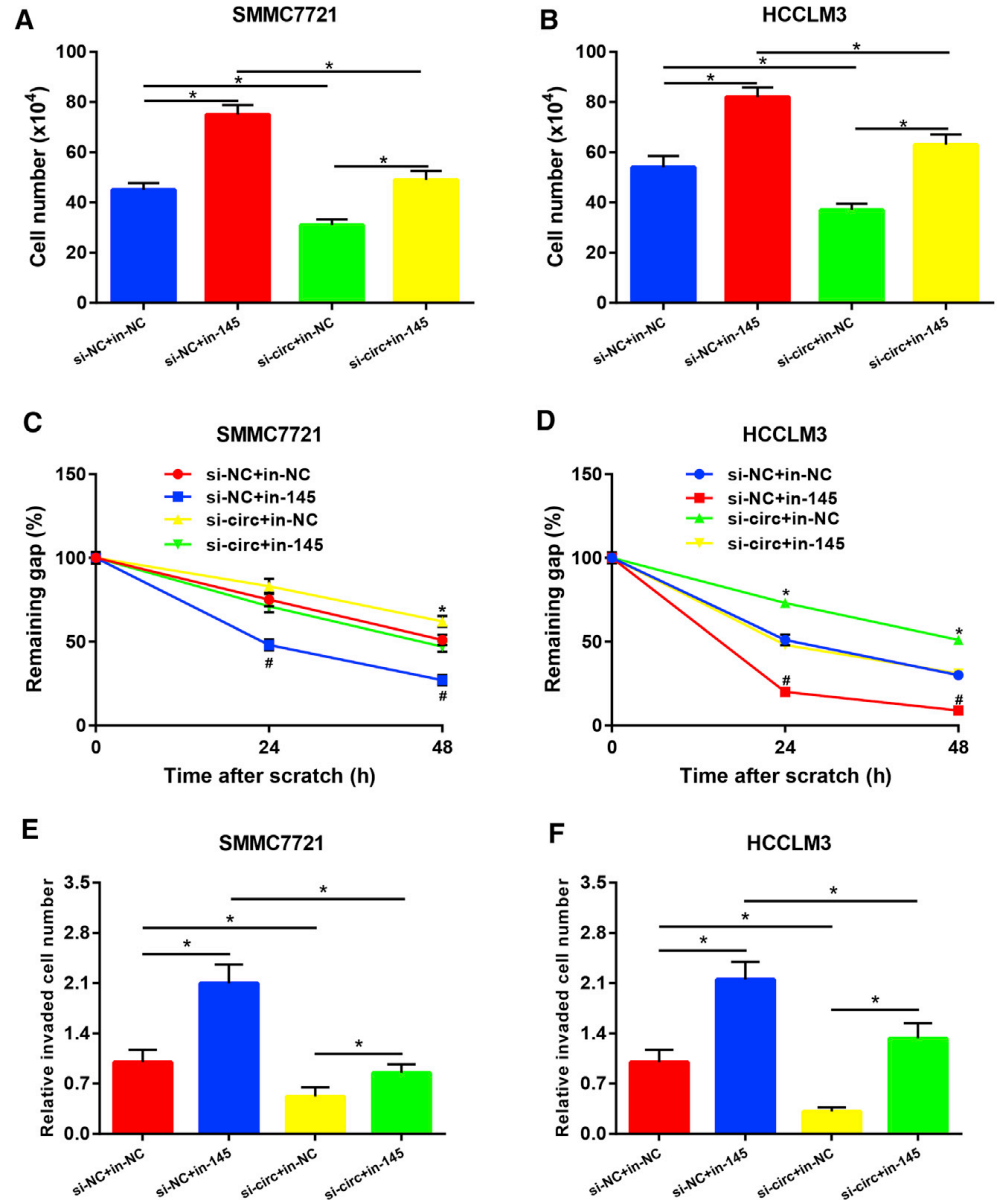
miR-145-5p Rescued the Oncogenic Roles of hsa_circ_0001955 in HCC (A) Knockdown of miR-145-5p rescued the effect of hsa_circ_0001955 on SMMC7721 growth. (B) Knockdown of miR-145-5p rescued the effect of hsa_circ_0001955 on HCCLM3 growth. (C) Knockdown of miR-145-5p rescued the effect of hsa_circ_0001955 on SMMC7721 migration. (D) Knockdown of miR-145-5p rescued the effect of hsa_circ_0001955 on HCCLM3 migration. (E) Knockdown of miR-145-5p rescued the effect of hsa_circ_0001955 on SMMC7721 invasion. (F) Knockdown of miR-145-5p rescued the effect of hsa_circ_0001955 on HCCLM3 invasion. ∗p < 0.05 (si-NC+in-NC compared with si-NC+in-145); ^#^p < 0.05 (si-circ+in-NC compared with si-circ+in-145).

In this part, we aimed to study the role of miR-145-5p and its effect on hsa_circ_0001955-mediated oncogenic functions in HCC. First of all, miR-145-5p expression in HCC was determined by qRT-PCR. As presented in Figures 5A and 5B, the expression levels of miR-145-5p in HCC cell lines and tissues were significantly lower than that in normal liver cell lines and matched normal tissues, which was identical with our previous analytic results from TCGA data. Next, the expression correlation of miR-145-5p with hsa_circ_0001955 was also assessed in 60 HCC samples (Figure 5C). The result indicated that hsa_circ_0001955 expression was significantly negatively correlated with miR-145-5p expression in HCC (R = −0.2829 and p = 0.0285). miR-145-5p mimic and inhibitor were implied to increase and decrease miR-145-5p expression in SMMC7721 and HCCLM3 cells (Figure 5D). Intriguingly, upregulation and downregulation of miR-145-5p expression respectively suppressed and promoted HCC cell growth (Figure 5E), migration (Figures 5F and 5G), and invasion (Figures 5H and 5I). Moreover, after inhibition of miR-145-5p, the effects of si-hsa_circ_0001955#3 on growth (Figures 6A and 6B), migration (Figures 6C and 6D), and invasion (Figures 6E and 6F) of HCC cells were partially rescued. These findings indicated that miR-145-5p played tumor-suppressive roles in HCC and rescued oncogenic effects of hsa_circ_0001955 in HCC.

### NRAS Was a Downstream Target of the hsa_circ_0001955/miR-145-5p Axis in HCC

**Figure 7:**
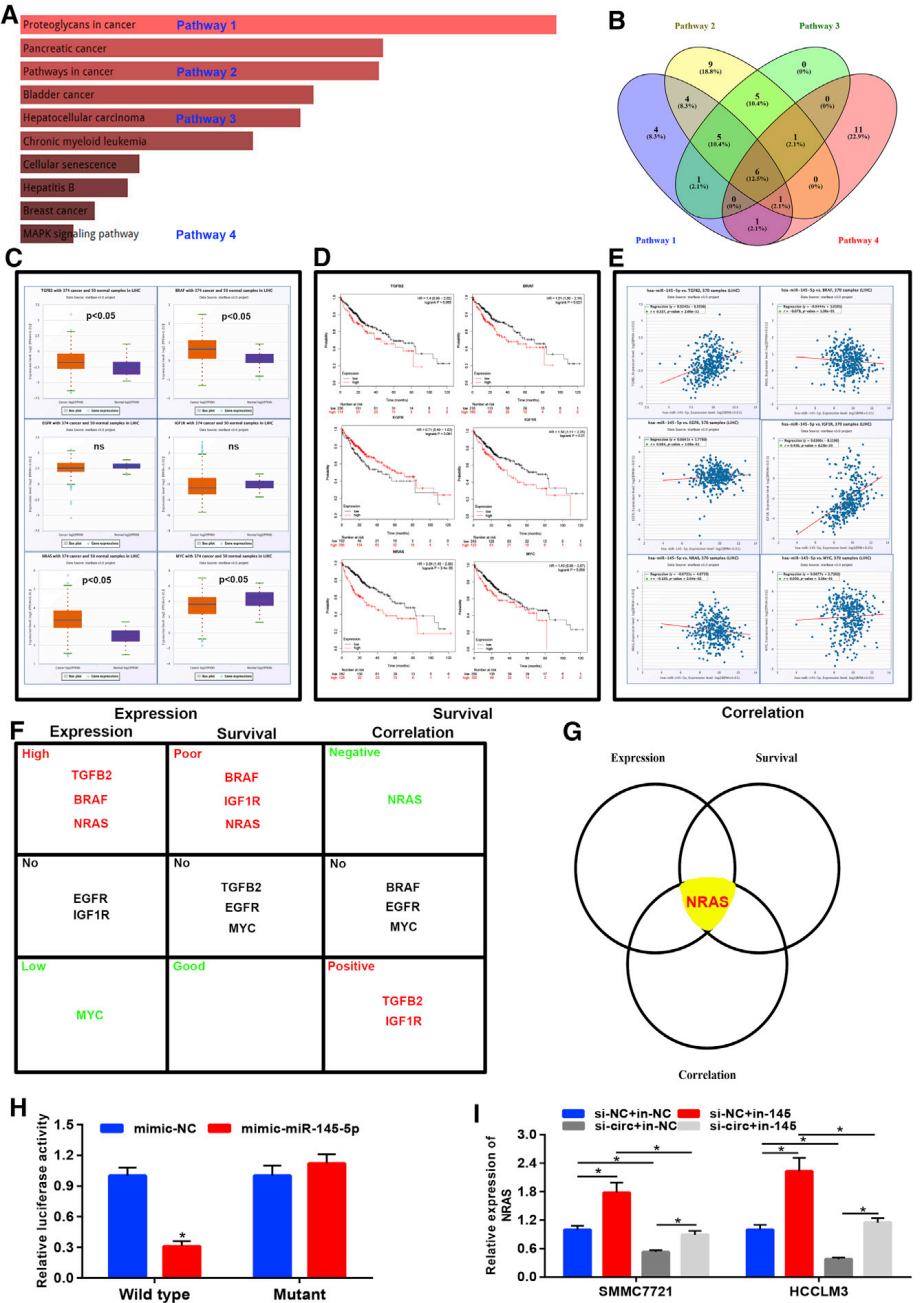
NRAS Was a Direct Target of the hsa_circ_0001955/miR-145-5p Axis in HCC (A) The top 10 enriched KEGG pathways of target genes of miR-145-5p. (B) The intersection of genes enriched in four selected pathways. (C) The expression levels of six genes in HCC. (D) The prognostic values of six genes in HCC. (E) The expression correlation between miR-145-5p and six genes in HCC. (F) The description of expression, survival, and correlation analyses for the six genes in HCC. (G) NRAS was identified as the potential target of miR-145-5p based on the analytic results from expression, survival, and correlation analyses. (H) The direct relationship between miR-145-5p and NRAS was determined by dual-luciferase reporter assay. (I) The expression change of NRAS after alteration of hsa_circ_0001955 and miR-145-5p. ∗p < 0.05. ns, represented 99999no significant difference.

To further explore the molecular mechanism of the hsa_circ_0001955/miR-145-5p axis in HCC, we predicted the target genes of miR-145-5p by miRNet, a comprehensive database for miRNA-associated studies. A total of 238 target genes were found as listed in Table S4. Next, these target genes were typed into Enrichr to perform Kyoto Encyclopedia of Genes and Genomes (KEGG) pathway enrichment analysis. The top 10 enriched KEGG pathways were automatically shown on the webpage and directly downloaded as depicted in Figure 7A. Four pathways of interest, containing proteoglycans in cancer, pathways in cancer, HCC pathway, and MAPK signaling pathway, were included for identifying the most potential target genes of miR-145-5p. We noticed that six genes (TGFB2, BRAF, EGFR, IGF1R, NRAS, and MYC) commonly appeared in the four included pathways (Figure 7B). According to the miRNA action mechanism, the potential targets of miR-145-5p should be oncogenes in HCC. The expression of six genes in HCC were assessed using starBase and showed that only TGFB2, BRAF, and NRAS expression levels were significantly upregulated in HCC (Figure 7C); survival analysis for the six genes revealed that high expression of three genes (BRAF, IGF1R, and NRAS) possessed poor prognosis of patients with HCC (Figure 7D); expression correlation analysis demonstrated that, among the six genes, only NRAS expression was negatively correlated with miR-145-5p expression in HCC (Figure 7E). The detailed description of expression, survival, and correlation analysis for TGFB2, BRAF, EGFR, IGF1R, NRAS, and MYC in HCC was presented in Figure 7F, suggesting that NRAS was the most potential target of miR-145-5p in HCC (Figure 7G). Dual-luciferase reporter assay also validated the direct binding of miR-145-5p with NRAS (Figure 7H). Moreover, the miR-145-5p inhibitor significantly increased NRAS expression, and this effect could be reversed after transfection with si-hsa_circ_0001955 (Figure 7I). Taken together, NRAS was the downstream target of the hsa_circ_0001955/NRAS axis in HCC.

## Discussion

circRNAs, ubiquitous endogenous RNAs, are found in most organisms. Despite circRNAs having been widely investigated during recent years, the function and mechanism of them in human cancers are still not well understood. In this study, we first focused on the expression levels of circRNAs in HCC. By intersection of DECs of three HCC circRNA microarray profiles from the GEO database (GEO: GSE78520, GSE94508, and GSE97332), we identified 21 circRNAs that were commonly upregulated in HCC in all three datasets. Some of them have been found to act as key players in cancer initiation and progression. For example, Bian et al.^16^ indicated that hsa_circ_0072088, also known as hsa_circRNA_103809, regulated proliferation and migration of colorectal cancer via the miR-532-3p/FOXO4 axis; Deng et al.^17^ confirmed that hsa_circ_0009910 promoted carcinogenesis of osteosarcoma by sponging miR-449a to increase interleukin-6R (IL-6R) expression. To preliminarily explore the function of these circRNAs in HCC, we further screened the top three most upregulated DECs in GEO: GSE78520, GSE94508, and GSE97332, and we noticed that only hsa_circ_0001955 commonly appeared in all three DEC sets from GEO: GSE78520, GSE94508, and GSE97332. hsa_circ_0001955 has been reported to be differentially expressed between triple-negative and luminal A subtypes of breast cancer and may be involved in the carcinogenesis of breast cancer.^18^ However, its function and mechanism in breast cancer and other human cancers, including HCC, have not been investigated. Consequently, we intended to ascertain whether hsa_circ_0001955 exerted oncogenic or tumor-suppressive roles in HCC.

First of all, HCC cell lines and clinical tissue samples were employed to validate the expression of hsa_circ_0001955. The results showed that hsa_circ_0001955 expression was exactly increased in HCC, which was inconsistent with the previous analytic results from circRNA microarrays. Moreover, clinical data analysis revealed that hsa_circ_0001955 expression was significantly correlated with large tumor size and advanced TNM stage. These findings indicated that hsa_circ_0001955 might act as an oncogenic circRNA in HCC. Next, functional experiments, containing cell counting assay, wound healing assay, and transwell invasion assay, were performed. Knockdown of hsa_circ_0001955 markedly inhibited cell proliferation, migration, and invasion, whereas overexpression of hsa_circ_0001955 led to the opposite effects.

To elucidate the detailed molecular action mechanism of hsa_circ_0001955, we first predicted its potential binding miRNAs because most functional circRNAs were found to serve as miRNA sponges to regulate gene expression and exert their functions.^19^ Four potential miRNAs (miR-1252-5p, miR-1296-5p, miR-145-5p, and miR-516a-5p) of hsa_circ_0001955 were predicted by starBase and CRI databases. By combination of the analytic results from expression and survival analyses for the four miRNAs in HCC, miR-145-5p was identified as the most potential miRNA of hsa_circ_0001955, later confirmed by a dual-luciferase reporter assay. Previous studies reveal that miR-145-5p was a tumor-suppressive miRNA in multiple cancers. For example, the suppressive effect of miR-145-5p on HCC growth has been reported by the team of Noh et al.^20^ Our functional assays also validated that miR-145-5p played negative roles in proliferation, migration, and invasion of SMMC7721 and HCCLM3 cells.

Finally, we further explored the downstream targets and pathways of hsa_circ_0001955/miR-145-5p in HCC. The predicted target genes of miR-145-5p were significantly enriched in cancer-associated pathways, such as proteoglycans in cancer^21^ and the MAPK signaling pathway.^22^ Six target genes of interest were selected for expression, survival, and correlation analyses, and the analytic results showed that NRAS, an oncogene encoding a membrane protein, was the most potential target of miR-145-5p. The direct binding of miR-145-5p and NRAS was also confirmed by a dual-luciferase reporter assay. Furthermore, NRAS expression was dramatically upregulated after knockdown of miR-145-5p, and this effect could be reversed by hsa_circ_0001955. We therefore concluded that hsa_circ_0001955 increased NRAS expression by regulating miR-145-5p, and thus facilitated proliferation, migration, and invasion of HCC. However, many more lab experiments and clinical trials need to be conducted to further validate these findings in the future.

In summary, we showed that hsa_circ_0001955 expression in HCC was significantly increased and its upregulation correlated with HCC progression. We also elucidated a positive regulatory role of hsa_circ_0001955 in HCC proliferation, migration, and invasion by influencing the miR-145-5p/NRAS axis. Collectively, targeting hsa_circ_0001955/miR-145-5p/NRAS may represent a potential therapeutic measure for patients with HCC.

## Materials and Methods

### Microarray Selection and Differential Analysis

In this study, we wanted to find the potential functional circRNAs in HCC. GEO (https://www.ncbi.nlm.nih.gov/geo/) is one of the most widely used public databases all over the world. Only the datasets about circRNA expression in human HCC tissue level were included. Finally, three datasets, containing GEO: GSE78520, GSE94508, and GSE97332, were selected for differential expression analysis. All three datasets were based on the platform of GPL19978 Agilent-069978 Arraystar Human CircRNA microarray V1. GEO: GSE78520 included three HCC cancer and three adjacent normal samples, GEO: GSE94508 included five HCC cancer and five adjacent normal samples, and GEO: GSE97332 included seven HCC cancer and seven adjacent normal samples. GEO2R, an online tool provided by the GEO database, was employed to perform differential expression analysis for the three datasets. |FC| ≥ 2 and p < 0.05 were set as the criteria to identify significant DECs.

### Cell Lines and Clinical Samples

All cell lines used in this study were purchased from the cell bank of the Chinese Scientific Academy. QSG7701, HL7702, Bel7402, HCCLNB, Huh7, HepG2, and HCCLM3 were maintained in DMEM medium (GIBCO, Life Technologies, CA, USA), and SMMC7721 was cultured in RPMI 1640 medium (GIBCO, Life Technologies, CA, USA) supplemented with 10% fetal bovine serum (FBS; Biological Industries, CT, USA) at 37 °C and 5% CO_2_ atmosphere. Fresh HCC tissues and matched adjacent normal tissues were obtained from 60 patients who received surgery from 2017 to 2018 at the First Affiliated Hospital of Zhejiang University School of Medicine (Hangzhou, China). The fresh tissues were quickly frozen and stored in liquid nitrogen until usage. This study was approved by the Ethics Committee of the First Affiliated Hospital of Zhejiang University School of Medicine. The written informed consent was obtained from each individual patient.

### qRT-PCR Analysis

Total RNAs from cells and tissues were first extracted using RNAiso plus Reagent (TaKaRa, Kusatsu, Japan). RNAs were reverse transcribed into cDNA, and qRT-PCR analysis was conducted as we previously described.^23^, ^24^, ^25^ The sequences of primers used in this study are as follows: hsa_circ_0001955 forward primer (5′-GCCTCTTCGAAATCAGGTGAA-3′) and hsa_circ_0001955 reverse primer (5′-CCATTCGAGAAAGCAGCTGG-3′); NRAS forward primer (5′-TGAGAGACCAATACATGAGGACA-3′) and NRAS reverse primer (5′-CCCTGTAGAGGTTAATATCCGCA-3′); GAPDH forward primer (5′- TGCACCACCAACTGCTTAGC-3′) and GAPDH reverse primer (5′-GGCATGGACTGTGGTCATGAG-3′). The expression level circRNA/gene (relative to GAPDH) or miRNA (relative to U6) was calculated by the method of 2^−ddCt^.

### Cell Transfection

The siRNAs targeting hsa_circ_0001955 and its negative control, hsa_circ_0001955 overexpression plasmid and its negative control, and the mimic and inhibitor of miR-145-5p and their corresponding negative control were purchased from Ribobio (Guangzhou, China). These reagents mentioned above were transfected into HCC cells using Lipofectamine 3000 in accordance with the manufacturer’s instructions. After 12 h post-transfection, culture medium was replaced with fresh medium. The target sequences of siRNAs were hsa_circ_0001955#1 (5′-TTCGAAATCAGGTGAAGGT-3′), hsa_circ_0001955#2 (5′-GAAATCAGGTGAAGGTCTC-3′), and hsa_circ_0001955#3 (5′-CTCTTCGAAATCAGGTGAA-3′).

### Cell Counting Assay

Cell proliferation was evaluated by cell counting assay. First, cells were transfected as described. At 12 h post-transfection, 10 × 10^4^ cells were re-seeded into six-well plates and cultured for 3 days. Cells in each well were counted.

### Wound Healing Assay

A total of 50 × 10^4^ pre-transfected cells were re-plated into six-well plates. When cells grew to 100% confluence, wound heal was made by a micropipette tip. Photographs were taken using microscopy at 0, 24, and 48 h.

### Transwell Invasion Assay

Cell invasion was assessed through transwell invasion assay. The inserts were first coated with Matrigel (BD Biosciences, USA). As a chemoattractant, 0.6 mL medium with 15% FBS was added into the lower compartment. Then, 0.2 mL serum-free medium containing 10 × 10^4^ pre-transfected cells was added into the pre-coated upper inserts of 24-well transwell chambers (Corning, USA). After incubation for 48 h, cells on the upper surface of the membrane were removed, and cells on the lower surface were fixed with 100% methanol for 15 min and stained with 0.1% crystal violet for 20 min. Finally, five random fields of each insert were obtained using a microscope (Olympus, Japan).

### miRNA Prediction

miRNAs that potentially bind to hsa_circ_0001955 were predicted using two databases, starBase (http://starbase.sysu.edu.cn/) and CRI (https://circinteractome.nia.nih.gov/). The intersected miRNAs from the two databases were identified using VENNY 2.1 (https://bioinfogp.cnb.csic.es/tools/venny/index.html) and were chosen for subsequent analysis.

### miRNA and Gene Expression Analysis

Expression levels of potential miRNAs of hsa_circ_0001955 and target genes of miR-145-5p were determined by starBase using TCGA HCC and normal liver data. A p value <0.05 was regarded as statistically significant.

### Kaplan-Meier Plotter Analysis

The prognostic values of potential miRNAs of hsa_circ_0001955 and target genes of miR-145-5p in HCC were assessed by Kaplan-Meier plotter (http://kmplot.com/analysis/), which is capable of accessing the survival effects of gene and miRNA from 20 various types of cancer. Genes and miRNAs were first entered into the Kaplan-Meier plotter. Then, survival plots were automatically generated, and log rank p value, hazard ratio, and 95% confidence interval were calculated and displayed on the webpage. Log rank p < 0.05 was considered as statistically significant.

### miRNet Analysis

miRNet (https://www.mirnet.ca/), a comprehensive database and analytic platform to dissect miRNA-target interactions and functional associations through network-based visual analysis, was employed to predict potential target genes of miR-145-5p. The miR-145-5p-target gene interactions were directly downloaded from miRNet.

### Dual-Luciferase Reporter Assay

The direct binding relationship between miR-145-5p and hsa_circ_0001955 or NRAS was determined using a dual-luciferase reporter assay. miR-145-5p mimic and wild-type/mutant reporter vectors were co-transfected into cells for 12 h using Lipofectamine 3000 according to the manufacturer’s instructions. After 12 h post-transfection, each well was replaced with fresh medium and incubated for another 24 h. Dual-luciferase Reporter Assay System Kit (017319; Promega) was introduced to measure the luciferase activity by a Varioskan Flash Spectral Scanning Multimode Reader (Thermo Scientific, Waltham, MA, USA). Firefly luciferase activity was normalized to Renilla luciferase.

### Statistical Analysis

The results were presented as mean ± standard deviation from at least three independent experiments. Differences between two groups were analyzed using Student’s t test. Chi-square test was utilized to estimate the correlation between circRNA expression and clinicopathological features. A p value < 0.05 was considered statistically significant.

## Author Contributions

W.L. and B.D. designed this work, performed experiments, analyzed data, and drafted the manuscript. W.F. revised the manuscript. All authors have read and approved the final version of the manuscript.

## Conflicts of Interest

The authors declare no competing interests.
